# Newly Developed System for the Robust Detection of *Listeria monocytogenes* Based on a Bioelectric Cell Biosensor

**DOI:** 10.3390/bios10110178

**Published:** 2020-11-17

**Authors:** Agni Hadjilouka, Konstantinos Loizou, Theofylaktos Apostolou, Lazaros Dougiakis, Antonios Inglezakis, Dimitrios Tsaltas

**Affiliations:** 1 EMBIO Diagnostics Ltd., Athalassas Avenue 8, Strovolos, Nicosia 2018, Cyprus; k.loizou@embiodiagnostics.eu (K.L.); theo.apo@embiodiagnostics.eu (T.A.); l.dougiakis@embiodiagnostics.eu (L.D.); a.inglezakis@embiodiagnostics.eu (A.I.); 2 Department of Agricultural Sciences, Biotechnology and Food Science, Cyprus University of Technology, 30 Archbishop Kyprianos, Limassol 3036, Cyprus; dimitris.tsaltas@cut.ac.cy

**Keywords:** *Listeria monocytogenes*, cell-based biosensor, bioelectric recognition assay, membrane-engineering

## Abstract

Human food-borne diseases caused by pathogenic bacteria have been significantly increased in the last few decades causing numerous deaths worldwide. The standard analyses used for their detection have significant limitations regarding cost, special facilities and equipment, highly trained staff, and a long procedural time that can be crucial for foodborne pathogens with high hospitalization and mortality rates, such as *Listeria monocytogenes*. This study aimed to develop a biosensor that could detect *L. monocytogenes* rapidly and robustly. For this purpose, a cell-based biosensor technology based on the Bioelectric Recognition Assay (BERA) and a portable device developed by EMBIO Diagnostics, called B.EL.D (Bio Electric Diagnostics), were used. Membrane engineering was performed by electroinsertion of *Listeria monocytogenes* homologous antibodies into the membrane of African green monkey kidney (Vero) cells. The newly developed biosensor was able to detect the pathogen’s presence rapidly (3 min) at concentrations as low as 10^2^ CFU mL^−1^, demonstrating a higher sensitivity than most existing biosensor-based methods. In addition, lack of cross-reactivity with other *Listeria* species, as well as with *Escherichia coli,* was shown, thus, indicating biosensor’s significant specificity against *L. monocytogenes*.

## 1. Introduction

Foodborne diseases are of great concern worldwide, as they cause thousands of deaths each year, as well as significant malfunctions in health care systems, national economies, and global trade. Bacteria, viruses, parasites, and chemicals that contaminate food at any stage of food production are the causative agents for these infections. It is estimated that 600 million people (i.e., 10% of the global population) get sick and 420,000 people die every year due to the consumption of contaminated food [1]. *Listeria monocytogenes* is an intracellular pathogenic bacterium widely distributed in the environment that has been determined as a causative agent of serious epidemic and sporadic food-borne illnesses in humans. Listeriosis can lead to gastroenteritis, meningitis, or other severe symptoms with high hospitalization and mortality rates (20–30%), especially in vulnerable populations. In 2016, listeriosis was the most severe illness with the highest hospitalization and mortality rate in Europe. In the meantime, it is estimated that 1600 people get listeriosis and about 260 people die each year in the USA [2].

Despite the intensified efforts to improve the hygiene conditions and prevent cross-contamination in production processes, it is difficult to eliminate *L. monocytogenes* from all products. Hence, food safety authorities have made pathogen detection a priority. Currently, the detection of *L. monocytogenes* in food is mainly performed following the ISO 11290-1:2017 [3]. This culture method is based on the pathogen’s physical and chemical characteristics and is a gold standard method, however, it is also time-consuming since it requires four days to provide the first indications for either presumptive presence or absence of the pathogen and five to seven days to undoubtedly confirm the pathogen’s presence. Furthermore, DNA analysis based on quantitative polymerase chain reaction (qPCR) or microarray methods have been developed. Even though these methods are precise, they have significant limitations regarding cost, special facilities, long procedural time, and highly trained staff requirements [4]. Rapid detection methods based on molecular techniques (e.g., real-time qPCR) and immunology-based methods (e.g., VIDAS (Vitek Immuno Diagnostic Assay System) for *L. monocytogenes*) have also been developed. However, molecular techniques are still restricted by high-cost equipment and reagents, as well as complicated nucleic acid extraction procedures, while immunology-based methods are restricted by lower detection sensitivity [5]. Developing rapid, low-cost methods with high sensitivity and specificity to detect bacteria in food and reduce the risk to public health remains a challenge.

Biosensors have drawn major interest as alternative methods for food contamination analysis. Their mode of action is based on their ability to detect specific targets and transform this information into a detectable signal [6]. According to the transducing elements, biosensors are classified as electrochemical, thermal, optical, and piezoelectric sensors, with a noteworthy rapid development of electrochemical biosensors for pathogen detection in the food and agricultural sector in the last decade [7,8,9,10]. According to the type of the biomolecule recognition element, biosensors are distinguished in enzyme-based (immobilized enzymes and proteins), microorganism-based biosensors; DNA biosensors (nucleic acids); immunosensors (an immobilized antibody or antibodies coupled with an enzyme or pigment); and cellular biosensors (immobilized cells or tissues) [10,11].

Living cell-based biosensors are techno-scientific systems that use cells as sensors to detect the status of the cellular environment and physiological parameters. The innovation of these biosensors is that, unlike other types of biosensors containing only living-extracted elements, they use live cells as receptors. This type of biosensor consists of the following: (a) living cells, the biological identification element, that acts as the primary signal transducer element and is used as the primary element for the collection and transmission of signals, and (b) the secondary transducer element that converts these responses in electrical signals. When cells interact with a stimulus, they produce changes in molecules or ions, changes in potential, or changes in impedance due to cell metabolism, etc. A secondary transducer element can detect these responses and convert them into electrical signals [12]. Cell-based biosensors have the advantages of increased stability and high biocatalytic activity, and their main feature is their ability to provide relevant cellular information in response to the sample and finally measure its activity. Furthermore, their robustness, high selectivity, specificity, and evolvability have raised cell-based biosensors as a significant revolution in analytical science [13]. However, only a few studies have been performed on *L. monocytogenes* detection using cell-based biosensors [14,15,16].

In combination with methods such as the BERA (Bioelectric Recognition Assay), the cell-based biosensors have been used in numerous environmental, chemical, and medical applications with remarkable results [17,18,19,20,21,22]. The BERA method is based on the insertion, by electroporation, of a large number of receptor molecules (antibodies, enzymes, etc.) on the cell membrane, increasing their selectivity for recognizing target analytes [23,24]. The methodology is based on measuring the change in membrane potential caused by the binding of the target molecule to the receptors previously inserted into the cell membrane. In the beginning, the cell membrane potential is stable due to the ions flowing through the ion channels. Subsequently, and after the target molecule binds to the receptor, its structure changes, resulting in its molecular charge being displaced within the cell membrane. As a result, a large number of ions concentrate on one side of the membrane. Opening the ion channel creates an ionic current that can be measured as a corresponding current.

In the present study, we report the development of a portable BERA-type sensor based on mammalian cells for the rapid detection of *L. monocytogenes*. The biosensor was developed performing a membrane-engineering process, where anti-L. monocytogenes antibodies were electroinserted in the membrane of the Vero cells to achieve a selective response against the pathogenic bacterium. The response was measured according to the principles of BERA [18,23,24,25,26] and results were obtained based on a novel sophisticated algorithm embedded in user-friendly software that connects via Bluetooth with an android device, thus, the end-user was instantly informed of the results of each analysis performed.

## 2. Materials and Methods

### 2.1. Materials and Reagents

Monkey African green kidney (Vero) cell cultures were provided from LGC Promochem (Teddington, UK). Fetal bovine serum (FBS), antibiotics (streptomycin-penicillin), L-glutamine and L-alanine, and trypsin/EDTA (ethylenediaminetetraacetic acid) were purchased from Sigma-Aldrich (Taufkirchen, Germany). Monoclonal antibodies against *L. monocytogenes* were purchased from antibodies-online.com and *L. monocytogenes* NCTC 11,994 and *L. innocua* NCTC 11,288 from Merck (Darmstadt, Germany). Sodium chloride was purchased from Merck (Darmstadt, Germany) and Brain Heart Infusion from Biolife (Milan, Italy).

### 2.2. Cell Culture and Antibody Electroinsertion

Cell culture was performed according to Apostolou et al. [26]. Briefly, Vero cells were cultured in Dulbecco’s medium with 10% fetal bovine serum (FBS), 10% antibiotics (streptomycin-penicillin), and 10% l-glutamine and l-alanine (nutrient medium). Cell detachment from the culture vessel was performed by adding trypsin/EDTA for 10 min at 37 °C and cells were collected by centrifugation (2 min/1200 rpm) at a final density of 2.5 × 10^6^ mL^−1^.

Membrane-engineered cells were created by electroinserting either the anti-*L. monocytogenes* p60 protein antibody clone p6007 (p60-biosensor) or the anti-*L. monocytogenes* actA antibody clone 3a15 (actA-biosensor) into the membrane of the Vero cells, based on a modified protocol of Zeira et al. [27]. In brief, cells were detached and collected after centrifuge (6 min/1000 rpm/25 °C). The cell pellet was resuspended in 400 μL PBS (phosphate-buffered saline) containing three different antibody concentrations (1, 5, and 10 μg mL^−1^) and incubated on ice for 20 min. After incubation, the cell-antibody mixture was transferred into electroporator cuvettes (4 mm) and electroinsertion was performed in the Eppendorf Eporator (Hamburg, Germany) by the application of two square electric pulses at 1800 V/cm. Subsequently, the mixture was transferred in a petri dish (60 × 15 mm^2^) containing 3 mL of nutrient medium and incubation took place at 37 °C and 5% CO_2_ for 24 h. Then, the medium was discarded from the petri dish and Vero/anti-L. monocytogenes cells were mechanically detached and collected with the nutrient medium in Eppendorf tubes.

It has been previously indicated that, based on the above electroinserting method, the membrane-engineered cells incorporate the specific antibodies in the correct orientation and show selective interaction against the target molecules [24,28].

### 2.3. Bacteria Culturing and Sample Preparation

*L. monocytogenes* NCTC 11994, *L. ivanovii* NCTC 11846, *L. innocua* NCTC 11288, and *Escherichia coli* ATCC 10,536 were used throughout this study. The strains were kept at −20 °C in nutrient broths supplemented with 50% glycerol, then, prior to their utilization, each strain was grown twice in Brain Heart Infusion broth at 37 °C for 24 h. Experimental assays were performed using overnight bacteria cultures (*L. monocytogenes*, *L. ivanovii*, *L. innocua*: 9 log CFU (colony forming units) mL^−1^ and *E. coli* 8 log CFU mL^−1^) that were centrifuged (10 min/3500 rpm/4 °C), washed twice with sterile saline solution, resuspended in the same diluent, and serially diluted to 2 log CFU mL^−1^. Then, diluents with the desired concentrations (2, 4, 6, and 9 log CFU mL^−1^ for *Listeria* species and 2, 4, 6, 8 log CFU mL^−1^ for *E. coli*) were tested with the newly developed biosensor system.

For bacteria counting, the overnight cultures were serially diluted with sterile saline solution. Then, triplicate diluents were surface plated in brain heart infusion (BHI) agar plates and incubation took place at 37 °C for 24 h. Subsequently, the colonies were counted, and the concentration of the bacteria was calculated in CFU mL^−1^.

### 2.4. Experimental Design and Assay Procedure

The present study was conducted in three experimental assays. The first assay was performed to compare and evaluate two different *Listeria monocytogenes* antibodies in the presence of the pathogen at various population levels. In the second assay, the effect of the concentration (1, 5, and 10 μg mL^−1^) of the electroinsterted antibody on the response of the membrane-engineered cells was investigated. Finally, in the third experimental assay, the cross-reactivity of the newly developed biosensor system was evaluated against *Escherichia coli* and other *Listeria* species.

**Figure 1:**
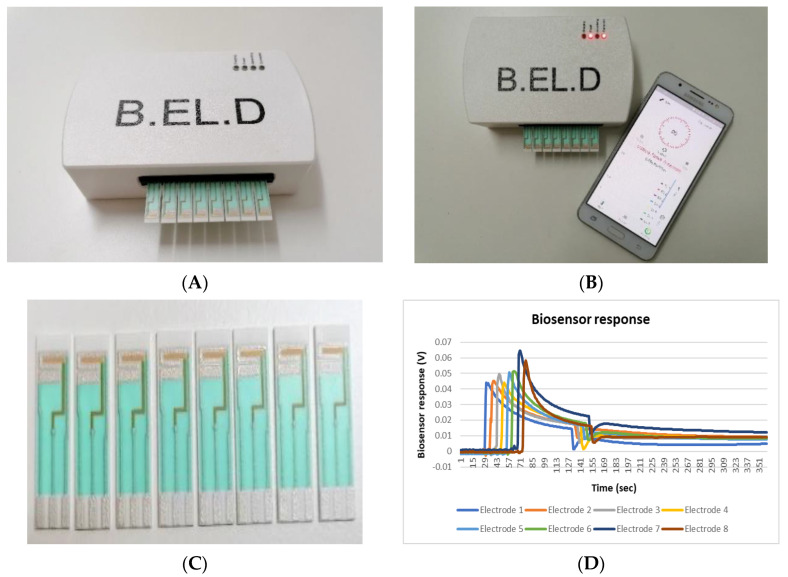
(**A**) Loading of the cells and samples on the electrode surface; (**B**) Results appear on the smartphone screen; (**C**) Screen-printed electrode sensors used for the assay, Zimmer and Peacock gold single electrodes; (**D**) Visualization of the electric signal via a voltage (Volts) vs. time graph.

All tests were conducted using a portable device developed by EMBIO DIAGNOSTICS (EMBIO DIAGNOSTICS Ltd., Cyprus). EMBIO’s device is a multichannel potentiometer with high accuracy A/D converters that measure electric signals from various biorecognition elements, such as cells. The studied elements are added on the surface of eight screen-printed electrodes (SPE) that are connected to the underside of a replaceable connector (Figure 1A). EMBIO’s device in combination with the bioelectric recognition assay (BERA), an established cell-based biosensor technology, has created a biosensor system that allows high throughput screening and rapid testing. Furthermore, the system connects via Bluetooth 4.0 with a smartphone or tablet, allowing the end-user to be instantly informed of each analysis result (Figure 1B). The system has been previously reported by Apostolou et al. [26]. The screen-printed electrodes were purchased from Zimmer and Peacock (Horten, Norway). Each single gold electrode was comprised of a 625 μm thick ceramic substrate (alumina) with three screen-printed electrodes (working electrode, reference electrode, and counter electrode). The working electrode was made of gold, and the reference electrode was made of silver/silver chloride. The counter electrode was canceled out by the measuring system (Figure 1C).

First, the samples (20 μL) were added on the top of each gold screen-printed electrode (first peak on the two-dimensional (2D) line graph) and the membrane-engineered cells (20 μL ≈ 5 × 10^4^ cells) were added after 60 s (120 values)(second peak on the 2D line graph).

The cell response was recorded as a time series of potentiometric measurements (in Volts), consisting of 360 values per sample. Each measurement lasted 3 min and the sampling rate was set at 2 Hz. After each measurement, cell responses were uploaded into a cloud server (Google Firebase) and calculations were conducted (Section 2.5), based on a newly developed algorithm that provided results regarding *Listeria monocytogenes* presence on the smartphone screen (Figure 1Β). Every single sample was tested eight times by using a set of eight individual sensors and each experiment was performed in duplicate.

One-hundred tests were initially conducted on sodium chloride solution (NaCl 0.85%), a diluent used for preparing microbial suspensions (blank samples). Subsequently, 100 tests of *L. monocytogenes* diluted in NaCl 0.85% at 4 different concentrations (2, 4, 6, and 9 log CFU mL^−1^) were performed. Results obtained from the biosensors (p60-biosensor and actA-biosensor with 1, 5, or 10 μg mL^−1^ antibody concentration) were evaluated and the biosensor with the best accuracy and performance characteristics was determined and used in the subsequent tests. Performance indices used for the evaluation were sensitivity (Se), specificity (Sp), positive predictive value (PPV), and negative predictive value (NPV). An explanation of these terms can be found in the respective reference [29]. Furthermore, a total of 150 tests were performed on *L. ivanovii*, *L. innocua*, and *Escherichia coli* broth samples to evaluate biosensor’s cross-reactivity with other bacteria. Finally, to demonstrate that the obtained response of the tested samples was associated with the presence of the electroinserted antibody, 40 tests (control, *L. monocytogenes*, *L. ivanovii*, *L. innocua*, and *Escherichia coli* samples) were conducted with non-engineered cells as well.

### 2.5. Algorithm for Response Processing

**Figure 2:**
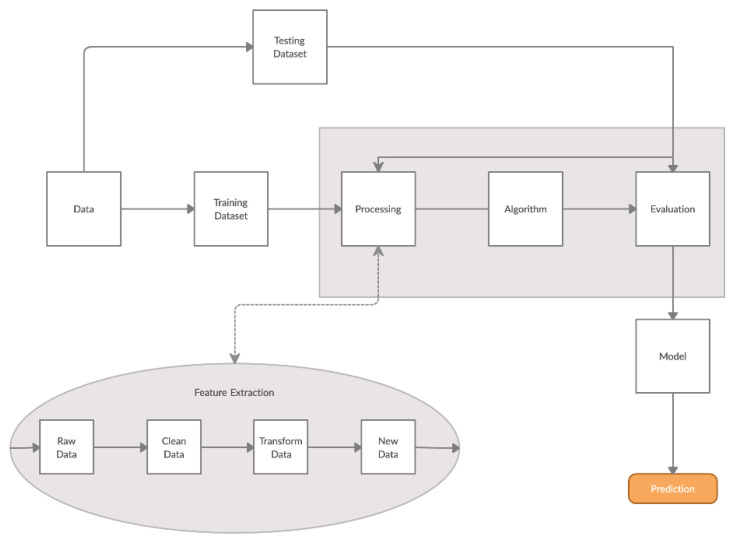
Bio Electric Diagnostics (B.EL.D) data analysis process on cloud functions. The analysis is being done in real time after the completion of the tests.

The process of the data analysis is summarized in Figure 2.

**Figure 3:**
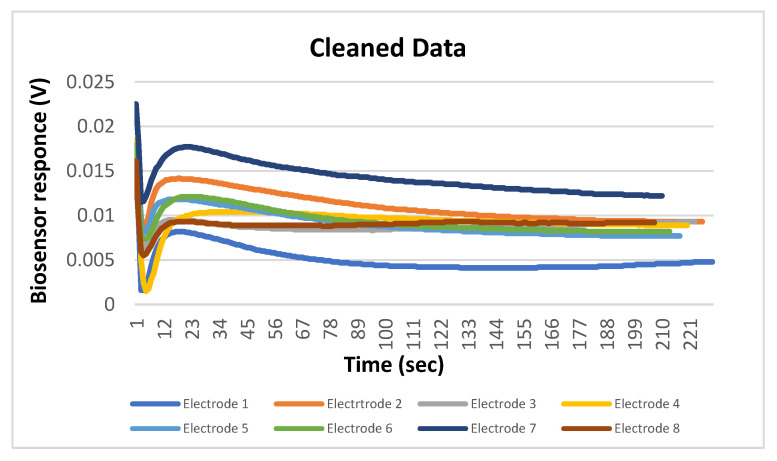
Visualization of the electric signal via a voltage (Volts) vs. time graph of the cleaned data.

**Figure 4:**
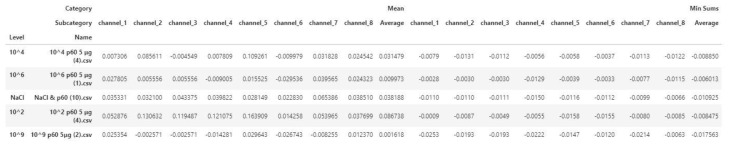
Feature vectors for five random samples (10^2^, 10^4^, 10^6^, 10^9^, and NaCl).

More analytically:

- Dataset: The dataset contained 200 measurements from 100 negative and 100 positive samples. Each measurement consisted of the data obtained by the 8 screen-printed electrodes that recorded the cell response as a time series of potentiometric measurements (in Volts) and comprised 360 values per electrode (sampling rate of 2 Hz). The detected measurements were visualized through a voltage/time graph (Figure 1D).
- Training/Testing Dataset: The obtained dataset was split into training and testing dataset as follows: 30% was used for training (60 measurements) and 70% was used for testing (140 measurements). The training dataset was utilized to determine the algorithm thresholds and the testing dataset was utilized for the algorithm evaluation.
- Processing/Feature Extraction: The processing of the dataset was performed in a two-step procedure. In the first step, peaks were determined and starting noise was cleaned to smooth and calibrate the signal before the first peak. Then, all values were shifted to start at y = 0 and from each experimental dataset values from 120 to 200 were shifted at x = 0 and kept for further analysis (Figure 3). In the second step, feature vectors were extracted from the cleaned data and used as input to develop an algorithm able to detect *L. monocytogenes* in sterile saline samples. Each feature vector was calculated based on the following: (a) the average values (mean) for each cleaned dataset and (b) the rolling average with rolling window size 50 (minimum sums) [30]. This procedure was applied in each electrode channel (channel 1, channel 2, etc.) and the overall test dataset (mean average and minimum sums average for all 8 electrodes) (Figure 4). Hence, from the initial experimental raw dataset with 360 × 8 values, only 1 × 18 (1 values for each channel (8 values in total) + 1 overall value/(a) and (b)) feature values were used for the sample discrimination and the algorithm development.

**Figure 5:**
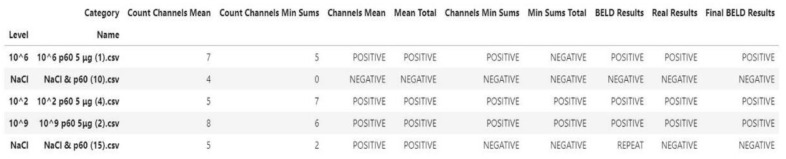
Calculation of the final results based on the feature vectors.

Algorithm: The algorithm used the feature vectors from the previous step as input to produce/calculate the final results. Two thresholds were set for mean values and minimum sums values and were compared to the respective values from each channel and from the average of the channels. Four primary results were extracted based on the following: (i) threshold comparing to each channel mean values, (ii) threshold comparing to the average mean value, (iii) threshold comparing to each channel minimum sums values, and (iv) threshold comparing to the average minimum sums values. In the case of each channel comparison (i and iii), the obtained result was the predominant one (e.g., if 5 electrodes gave ”positive” result and 3 gave ”negative”’ result based on the threshold, the result would be ”positive”). The final result was the predominant result obtained from the above calculations (e.g., if (i) channel mean values, ”positive”; (ii) average mean value, ”positive”, (iii) channel minimum sums values, ”negative”, and (iii) average minimum sums values: ”positive”, the final result would be ”positive”). In the case of two ”positive” and two ”negative”, the final result was the one obtaining from the average minimum sums value (Figure 5).

Evaluation: A testing dataset was used for the evaluation of the model. Different thresholds were tested and determination of the one providing the most accurate results was achieved.

### 2.6. Statiatical Analysis

One-way analysis of variance (ANOVA) was used to assess the statistical differences among the feature values obtained from the biosensors.

## 3. Results and Discussion

### 3.1. Antibody Selection and Biosensor Response in the Presence of L. monocytogenes

This study aimed to develop a biosensor that could perform rapid and robust detection of the pathogenic bacterium *L. monocytogenes*, using a modified protocol of a cell-based biosensor technology that has been previously reported by Apostolou et al. [26]. To develop the method, two different *Listeria monocytogenes* antibodies were initially used for the membrane engineering, creating two different biosensors. The two biosensors were created using either the anti-L. monocytogenes p60 protein antibody clone p6007 (p60-biosensor) or the anti-L. monocytogenes actA antibody clone 3a15 (actA-biosensor) at three different concentrations (1, 5, and 10 μg mL^−1^). The first antibody recognizes natural and recombinant *L. monocytogenes* extracellular p60 protein, an invasion associated protein (iap) essential for cell viability encoded by *iap* gene, and the second antibody recognizes *L. monocytogenes* protein actA, a surface protein necessary for intra- and intercellular motility that appears to be a multifunctional virulence factor. The *iap* and *actA* genes also exist in other *Listeria* species but their produced proteins differ from that of *L. monocytogenes* [31].

**Figure 6:**
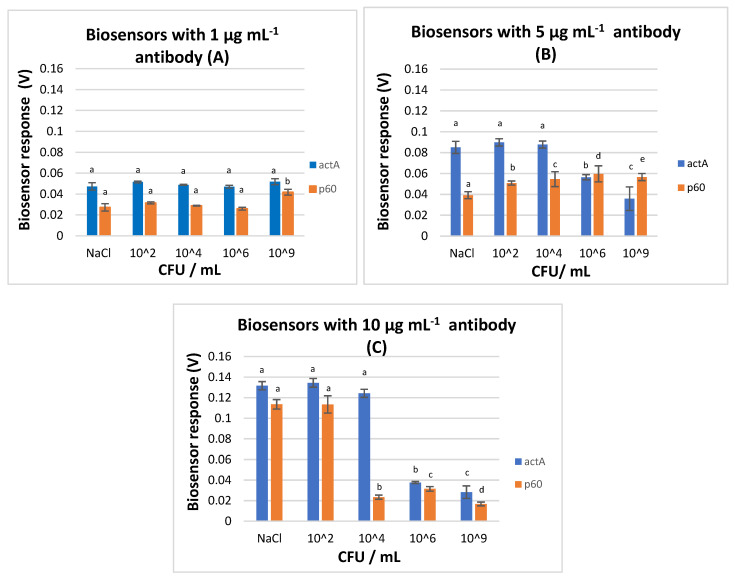
Response of actA and p60-biosensors with (**A**) 1 μg mL^−1^, (**B**) 5 μg mL^−1^, and (**C**) 10 μg mL^−1^ antibody concentration in the absence (NaCl 0.85%) and presence of *L. monocytogenes* at 4 different final concentrations (2, 4, 6, and 9 log CFU mL^−1^). Error bars represent the standard errors of the mean value of all replications. Columns marked with different letters indicate that response was significantly (*p* < 0.05) different from the respective of the blank samples (NaCl) for each experimental assay.

The results indicated that when the antibody concentration was at 1 μg mL^−1^, the actA-biosensor could not discriminate samples with and without the pathogen (0.047–0.051 mV), while the p60-biosensor was able to produce a significantly different signal only when the pathogen’s population was at 9 log CFU mL^−1^ (0.027–0.031 mV in NaCl, 10^2^, 10^4^, 10^6^ CFU mL^−1^, and 0.041 mV in 10^9^ CFU mL^−1^) (Figure 6A). More accurately, no pattern was visible for the different antibodies at this concentration, and this was attributed to the low number of the electoinserted antibodies into the membrane-engineered cells.

The signal obtained from the actA-biosensors with 5 and 10 μg mL^−1^ antibody concentration could distinguish blank samples from samples with *L. monocytogenes* at 6 and 9 log CFU mL^−1^ with values ranging from 0.035 to 0.056 mV and from 0.028 to 0.037 mV, respectively. Nevertheless, the biosensors were not able to discriminate blank samples from samples with *L. monocytogenes* at 2 and 4 log CFU mL^−1^, since similar voltage responses were observed (Figure 6B,C).

The best discrimination was achieved by p60-biosensor with 5 μg mL^−1^ antibody concentration. At this antibody concentration, increasing biosensor response was observed against the increasing *L. monocytogenes* population, with a 50% higher voltage response between the control and the inoculated samples with the higher bacterial concentrations (6 and 9 log CFU mL^−1^). Contrary to this, the p60-biosensor with 10 μg mL^−1^ antibody concentration could distinguish blank samples (0.116 mV) from samples with *L. monocytogenes* at 4, 6, and 9 log CFU mL^−1^, but not from samples with *L. monocytogenes* at 2 log CFU mL^−1^ (0.117 mV). Hence, it was demonstrated that the p60-biosensor with 5 μg mL^−1^ antibody concentration had the best discrimination ability, setting the method’s limit of detection at 10^2^ CFU mL^−1^. This is the lowest LOD being reported in studies that have developed various biosensors (optical, piezoelectric, amperometric, impedimetric, cell-based) for the detection of *L. monocytogenes* in culture and food samples [32,33,34,35,36].

From the conducted experiments, it was observed that the p60-biosensor with 5 μg mL^−1^ antibody concentration produced an increasing pattern against the increasing analyte (CFU mL^−1^). However, when the concentration of the antibody was augmented at a density of 10 μg mL^−1^, a decreasing pattern against the increasing analyte (CFU mL^−1^) was revealed. Although the sensitivity of a biosensor is largely dependent on the physical properties of the transducer system, the amount of the biological receptor molecules immobilized on the sensor surface remains an issue.

The binding of the antigens to antibodies adsorbed on a surface is a complex phenomenon and can be described as a two-step process. Firstly, the surface-adsorbed antibodies bind to the antigens through one of their fragment antigen-binding (F(ab)) domains. If they remain bounded, the second F(ab) domain has an opportunity to bind to any other antigen. Consequently, the equilibrium surface concentration of bounded IgG molecules, which increases with the surface concentration of the antigens, is the sum of two contributions. Therefore, antibodies which bound through a single F(ab) lead to a lower surface equilibrium.

De Michele et al. [37] recently, showed that the large size of IgGs played an instrumental role. Antigens diffusing in the bulk, see a few available IgGs on the surface which is reduced concerning the number of sites that are already occupied. In fact, a significant number of the unbounded antigens are unavailable due to the large size of IgGs, which overlap the unbounded sites of neighboring IgGs, thus, making them invisible for other antigens. In this way, antibodies that are immobilized onto the surfaces in a relatively higher density will induce locally crowded areas. Cho et al. [38] showed that such cluster formation could negatively affect the performance of the immunoassays, reporting that the binding capability was approximately 10 times lower than the maximum.

Additionally, Kwon et al. [39], in one of their studies, found that the immobilized antibodies that were active for binding antigens decreased from 150% to 60% as the density of immobilized antibodies was increased from 0.3 to 2.5 ng/mm^2^. In either case, the data clearly showed that as the density of the antibody increased, the steric accumulation of antigen-antibody complexes decreased the activity of the immobilized antibody. This demonstrates that the amount of antigen that binds depends on the density of the immobilized antibody.

Ideally, the antibody should specifically recognize and bind its antigen at the lowest possible concentration. Since the biosensor surfaces are in the μm scale, the optimum density of immobilized antibodies onto the surface result in enhanced detection sensitivity.

On the basis of the above, and since the p60-biosensor with 5 μg mL^−1^ antibody concentration had the best ability to separate blank from inoculated samples, especially when the pathogen was present at high concentrations, the p60 protein antibody clone p6007 was selected as the best antibody for the detection of the pathogen and the 5 μg mL^−1^ antibody concentration was selected as the optimum density for the *L. monocytogenes* biosensor.

#### Calculation of Method’s Performance Characteristics

As mentioned above (Section 2.5), 70% of the samples was used for testing (i.e., 140 samples, 77 positive and 63 negative). Parallel testing of the samples with the ISO and the newly developed method resulted in true-positive (TP), true-negative (TN), false-positive (FP), and false-negative (FN) results. On the basis of these results, the performance indices of the newly developed method were calculated and are summarized in the following Table 1:

Furthermore, the p60 biosensor with 5 μg mL^−1^ antibody concentration demonstrated perfect discrimination among samples with the highest inoculum level (9 log CFU mL^−1^) and the rest of the samples with 100% accuracy, sensitivity, specificity, positive predictive value, and negative predictive value.

### 3.2. Selectivity Assay

**Figure 7:**
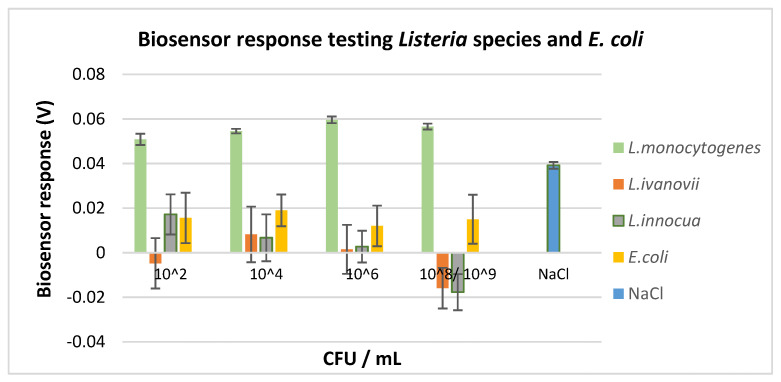
Response of p60-biosensor with 5 μg mL^−1^ antibody concentration at broth samples of *L. monocytogenes, L. ivanovii*, *L. innocua*, and *Escherichia coli* at four different population levels (*L. monocytogenes, L. ivanovii*, *L. innocua:* 2, 4, 6, and 9 log CFU mL^−1^; *E. coli*: 2, 4, 6, 8 log CFU mL^−1^).

The p60 protein that exists in other *Listeria* species differs from the *L. monocytogenes* p60 protein. However, *Listeria ivanovii*, a pathogenic bacterium with extremely rare cases of human disease, and *Listeria innocua*, a non-pathogenic bacterium widely distributed in the environment, were also included in the experimental assay to evaluate biosensor’s cross-reactivity with other *Listeria* species. In addition, the biosensor’s response in the presence of *Escherichia coli*, a microorganism that is often present in several food categories indicating faecal contamination and poor hygiene practices, was also evaluated. Evaluation of the biosensor’s cross-reactivity with *E. coli*, *L. ivanovii*, and *L. innocua* indicated that the *L. monocytogenes* p60-biosensor with 5 μg mL^−1^ antibody concentration was able to distinguish *L. monocytogenes* from the other *Listeria* species, as well as from *Escherichia coli*, infallibly. To evaluate biosensor’s selectivity, all bacteria were diluted in sterile saline solution and tested at the same concentration range (Figure 7). In every case examined, the biosensor’s response was statistically different (*p* < 0.05) from the observed response against the similar concentrations of *L. monocytogenes*.

More accurately, in the presence of *L. ivanovii* and *L. innocua*, the biosensor’s response was significantly different with substantial variations at every population level. The observed response was attributed to bacterial membrane potential dynamics [40]. Hence, it was indicated that the newly developed biosensor had no cross-reactivity with other *Listeria* species. A similar response was observed in the presence of *Escherichia coli*, thus, indicating no reaction between the samples and the biosensor. The response was less variable among different concentrations but significantly different (*p* < 0.05) to the ones observed for the similar concentrations of *L. monocytogenes* (Figure 7). Furthermore, the presence of *L. ivanovii*, *L. innocua,* and *Escherichia coli* revealed different cell responses as compared with the control values. However, the response was not a result of a specific reaction, since no detection pattern was demonstrated. Finally, based on the conducted experiments the non-engineered membrane cells seem to react in the presence of *L. monocytogenes*, *L. ivanovii*, *L. innocua*, and *Escherichia coli*, but the differences on response were statistically non-significant (data not shown). Hence, the biosensor’s selectivity assay was determined for *L. monocytogenes*. Contrary to these results, the p60 biosensor with 1 μg mL^−1^ antibody concentration was not able to distinguish samples with *L. monocytogenes* from samples with the other *Listeria* species (data not shown).

From the selectivity assay, it was validated that the bioelectric recognition assay was combined with the molecular identification through membrane engineering for the detection of the pathogenic bacterium *Listeria monocytogenes* with successful results. The presence of *L. monocytogenes* and attachment to its respective antibody caused a measurable change in the cell membrane structure that led to the discrimination between positive and negative samples. Hence, membrane engineering [19] was a critical step in the successful development of the biosensor system, since the electroinsertion of the anti-L. monocytogenes antibody on the surface of the Vero cells fortified the selectivity of the system against the pathogenic bacterium.

## 4. Conclusions

The present study demonstrates a rapid, high throughput, and portable screening system for *L. monocytogenes* detection, based on membrane-engineered cells. The newly developed biosensor system is combined with a sophisticated algorithm embedded in user-friendly software that allows the end-user to be instantly informed of the analysis results. The aim of this study was the proof-of-concept of the biosensor system for *L. monocytogenes* detection and the optimization of parameters that affect its performance, such as the type and the concentration of the electroinserted antibody. The newly developed biosensor was proven to be a robust and selective tool for *L. monocytogenes* detection, with a limit of detection as low as 10^2^ CFU mL^−1^. Therefore, in future research, the essay will be optimized in different food substrates and validation of its ability to detect pathogen’s presence on actual food samples will be performed, identifying and eradicating potential impediments due to the matrix effect.

## Figures and Tables

**Table 1:** Performance indices of the p-60 biosensor with 5 μg mL^−1^ antibody concentration.

Results ^1^		Performance Indices ^2^	
TP	75	Se	97.4%
FP	2	Sp	84.13%
TN	53	PPV	88.24%
FN	10	NPV	96.36%

^1^ TP, true positive; TN, true negative; FP, false positive; FN, false negative. ^2^ Se, sensitivity; Sp, specificity; PPV, positive predictive value; NPV, negative predictive value.
